# Maresins: Specialized Proresolving Lipid Mediators and Their Potential Role in Inflammatory-Related Diseases

**DOI:** 10.1155/2018/2380319

**Published:** 2018-02-20

**Authors:** Shi Tang, Ming Wan, Wei Huang, R. C. Stanton, Yong Xu

**Affiliations:** ^1^Endocrinology Department, The Affiliated Hospital of Southwest Medical University, Luzhou, Sichuan 646000, China; ^2^Endocrinology Department, The Affiliated Hospital of Nuclear Industry 416 Hospital, Chengdu, Sichuan 610000, China; ^3^Joslin Diabetes Center, Boston, MA, USA; ^4^Beth Israel Deaconess Medical Center, Boston, MA, USA; ^5^Harvard Medical School, Boston, MA, USA; ^6^Collaborative Innovation Center for Prevention and Treatment of Cardiovascular Disease of Sichuan Province, Southwest Medical University, Luzhou, Sichuan 646000, China

## Abstract

Acute inflammatory responses are host-protective and normally self-limited; these responses can maintain cell homeostasis and promote defense against various infections and damage factors. However, when improperly managed or inappropriately activated, acute inflammation can lead to persistent and uncontrolled chronic inflammation, which is associated with many other chronic diseases including cardiovascular disease and metabolic disease. Recently, studies have shown that resolution of acute inflammation is a biosynthetically active process. Specialized proresolving lipid mediators (SPMs) known as resolvins and protectins are autacoids that resolve inflammation. A new family of anti-inflammatory and proresolving lipid mediators have recently been reported, known as maresins, which are biosynthesized from docosahexaenoic acid (DHA) by macrophages, have a conjugated double-bond system, and display strong anti-inflammatory and proresolving activity. Here, we review the biological actions, pathways, and mechanisms of maresins, which may play pivotal roles in the resolution of inflammation.

## 1. Introduction

Acute inflammatory responses are defined as the activation of the innate immune system when the body is damaged or invaded by pathogens; leukocytes migrate from the circulation to the site of trauma or microbial invasion, forming inflammatory exudates and the release of inflammatory mediators of interleukin (interleukin, IL-1*β*, IL-6), tumor necrosis factor-*α* (TNF-*α*), high mobility group box-1 protein (HMGB1), prostaglandins, and so forth. This is followed by local vascular expansion, increase in permeability, leukocyte exudation, and, consequently, removal of pathogens. Inflammation is often accompanied by local painful swelling that is red and hot, along with other symptoms [1].

Proinflammatory cytokine production is a major feature of the inflammatory response. Often positive, the inflammatory response is temporary, only occurring locally, and is activated to fight invasion of pathogens and to promote repair of damaged tissue. However, when uncontrolled or inappropriately activated, acute inflammation can lead to persistent chronic inflammation, causing asthma and neurological degenerative disorders, as well as metabolic diseases, including diabetes, obesity, cardiovascular disease, and even cancer; if the inflammatory response is left unchecked, many inflammatory mediators are released into the blood, causing sepsis, which can lead to death [2]. Therefore, it is very important to regulate the inflammatory response at a clinical level.

Inflammation is an important defense mechanism of the host, which is driven not only by a series of proinflammatory mediators but also by a set of inflammatory self-limited mechanisms to regulate the development and resolution. Due to the these self-limited mechanisms, when inflammation has developed to an appropriate stage, the body produces endogenous proresolving lipid mediators, which remove inflammatory cells and proinflammatory mediators, repair damaged tissue, and terminate inflammatory responses in time [3, 4]. Therefore, insufficient secretion and/or dysfunction of proresolving lipid mediators do not allow the timely resolution of inflammation, which then progresses to chronic inflammation [5].

Resolution of inflammation is an active and highly regulated cellular and biochemical process [6]. Timely resolution of inflammation is crucial for preventing severe and chronic inflammation. Recently, several endogenous proresolving lipid mediators have been discovered, including lipoxins, resolvins, protectins, and maresins, which are heavily involved in driving inflammatory resolution and successfully terminating inflammation [7, 8]. Hence, specialized proresolving lipid mediators are a new focus for research. Many studies have shown the benefits of these lipid mediators that can limit tissue infiltration of polymorphonuclear leukocytes (PMNs), reduce collateral tissue damage by phagocytes, shorten the resolution interval (Ri), enhance macrophage phagocytosis and efferocytosis, and counterregulate proinflammatory chemical mediators [9].

## 2. Synthesis and Classification of Maresins

The omega-3 fatty acids eicosapntemacnioc acid (EPA) and DHA, which are found in fish oils, have long been known to be important for maintaining organ function and health, as well as reducing the incidence of inflammation [10, 11]. Maresins (*ma*crophage mediators in *re*solving *in*flammation) are derived from the omega-3 fatty acid DHA [12]. Maresins are produced by macrophages via initial lipoxygenation at the carbon-14 position by the insertion of molecular oxygen, producing a 13S,14S-epoxide-maresin intermediate that is enzymatically converted to maresin family members maresin 1, maresin 2, and maresin conjugate in tissue regeneration (MCTR) [9] (Table 1).

Maresin 1 was the first maresin to be identified [12]. Biosynthesis of maresin 1 in macrophages involves initial oxygenation of DHA with molecular oxygen, followed by epoxidation of the 14-hydroperoxy-intermediate that is subsequently converted to 13S,14S-epoxy-maresin. The complete stereochemistry of this epoxide intermediate is 13S,14S-epoxy-docosa-4Z,7Z,9E,11E,16Z,19Z-hexaenoic acid [13]. This epoxide intermediate is then proposed to be enzymatically hydrolyzed via an acid-catalyzed nucleophilic attack by water at carbon-7, resulting in the introduction of a hydroxyl group at that position and double-bond rearrangement to form the stereochemistry of bioactive maresin 1 [14].

However, when the 13S,14S-epoxy-maresin intermediate is followed by conversion via soluble epoxide hydrolase (sEH), it is then converted to additional bioactive products by human macrophages. Here, we nominated the new bioactive macrophage product as maresin 2 [15].

Recently, a new series of bioactive peptide-lipid-conjugated mediators that are produced during the later stages of self-resolving infections have been uncovered [16]. Researchers identified these mediators from human milk, mouse exudates, and human macrophages [17], and they cause lipoxygenation of DHA, producing a maresin-epoxide intermediate that is converted to sulfido-conjugate (SC) with triene double bonds, which belongs to the maresin family. Given that their production was initiated by oxygenation at carbon-14, these mediators were named maresin conjugates in tissue regeneration (MCTRs) [18].

## 3. Key Biosynthesis Enzymes of Maresins

**Figure 1:**
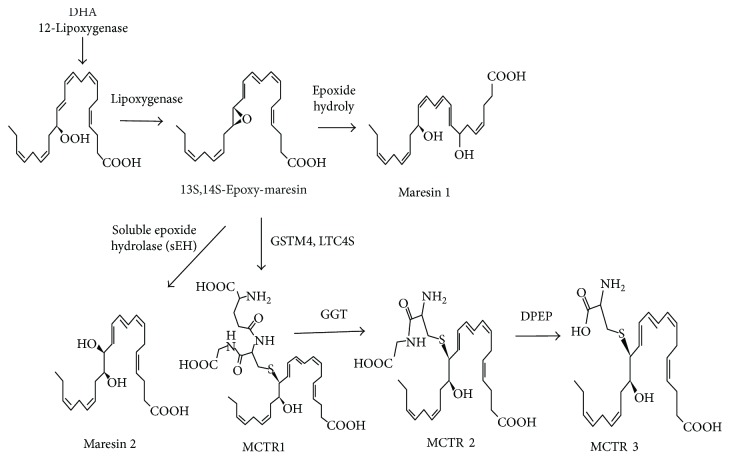
Maresin biosynthetic pathway [48].The pathway is initiated by the lipoxygenation of DHA to yield 13S,14S-epoxy-maresin. This intermediate is then enzymatically hydrolyzed to maresin 1 or via a soluble epoxide hydrolase (sEH) to maresin 2. 13S,14S-epoxy-maresin is also a substrate for glutathione S-transferase MU 4 (GSTM4) and leukotriene C4 synthase (LTC4S) yielding MCTR1, which is then converted to MCTR2 by gamma-glutamyl transferase (GGT) and to MCTR3 by dipeptidase (DPEP).

Human macrophage 12-lipoxygenase (12-LOX) initiates biosynthesis of maresins and, more importantly, is responsible for producing 13S,14S-epoxy-maresin [15] (Figure 1). Activation of 12-LOX in macrophages oxidizes DHA at carbon-14 sites in the major S-configuration and is also involved in the conversion of the 14-hydroperoxy group of 4Z,7Z,10Z,12E,16Z,19Z-docosahexaenoic acid to the 13S,14S-epoxide intermediate process, showing cyclooxygenase activity, manifested as alcohol capture [19]. 12-LOX also catalyzes the formation of lipoxins by leukotriene A4 (LTA4), which is susceptible to epoxide inhibition, for example, LTA4 or 13S,14S-epoxide intermediates [20]. Interestingly, the 13S,14S-epoxide intermediates only inhibit the conversion of 12-LOX to arachidonic (eicosatetraenoic) acid and do not play a role in DHA conversion, suggesting that 13S,14S-epoxide intermediates can exert a positive feedback on the maresin synthesis pathway and enhance resolution of the inflammation [19]. In addition, the level of messenger RNA expression of 12-LOX was shown to remain unchanged during differentiation of human monocytes to macrophages (M0, M1, and M2) [15].

Studies have shown that the biosynthesis of maresin 2 relates to the mammalian sEH protein (Figure 1); mammalian sEH enzymes catalyze the hydrolysis of a broad category of epoxides, including epoxyeicosatrienoic acids, LTA4, and even hepoxilins [15, 21]. sEH enzymes are active in mononuclear cells and macrophages [22, 23].

In the proposed MCTR biosynthetic pathway, human macrophage 12-LOX is the initiating enzyme, converting docosahexaenoic acid to 13S,14S-epoxide intermediates, which is converted to MCTR1 by leukotriene C4 synthase (LTC4S) and catalyzed glutathione S-transferase MU 4 (GSTM4). Both of these enzymes expressed in human macrophages and catalyze the conversion of LTA4 to leukotriene C4 (LTC4), which displays potent vasoactive and smooth muscle constricting actions. What is interesting is that GSTM4 gave higher affinity to 13S,14S-eMaR, whereas LTC4S has a higher affinity to LTA4. This quality may determine the balance between the LTC4 and the MCTR1. MCTR1 is the proposed precursor to MCTR2 and MCTR3, and gamma-glutamyltransferase (GGT) converts MCTR1 to MCTR2, which is then converted to MCTR3 by a dipeptidase (DPEP) enzyme (Figure 1). Both of the enzymes participate in the cysteinyl leukotriene pathway, and the GGT enzyme gave higher affinity to MCTR1 than LTC4. Their relative expression at sites of inflammation may lead to different disease processes; they also provide targeted therapeutic strategies to upregulate SPM formation [24]. However, the mechanism of maresins and their receptors is not clear, and thus, additional experiments are needed to investigate further.

## 4. Biological Actions of Maresins

Acute inflammation can lead to persistent and uncontrolled chronic inflammation, which can lead to severe diseases such as lung disease, vascular disease, and metabolic disease [25, 26].Currently, antibiotics are still the main treatment of acute infection following clinical diagnosis. However, with the serious threat of emerging pathogens, especially antibiotic-resistant ones, it is imperative to research and develop new therapeutic interventions of increasing the host anti-infective mechanisms [27].

Inflammatory resolution has become a new focus of inflammation research, and specialized proresolving lipid mediators have become a new strategy for inflammatory therapy [9]. The synthesis of anti-inflammatory drugs with endogenous anti-inflammatory mediators has important clinical significance. Studies have shown that targeted intervention with specialized proresolving lipid mediators can reduce the use of antibiotics for treating infection in the host reaction process, thus providing a new way to seek and develop more effective antimicrobial therapies [28].

**Figure 2:**
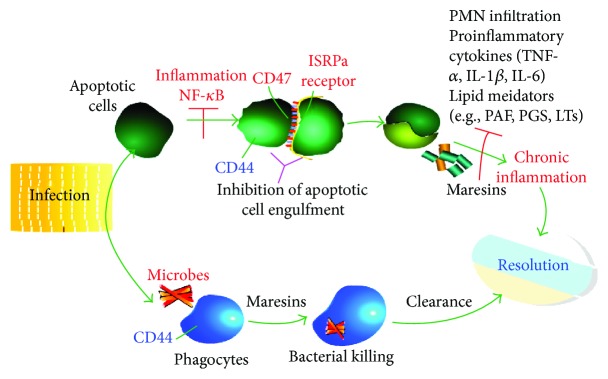
Maresins in the resolution pathway. Maresins stimulate efferocytosis and the uptake of debris for successful clearance from tissues and resolution. Maresins block NF-*κ*B and counterregulate proinflammatory mediators and lipid mediators; inhibition of containment of apoptotic cells leads to chronic inflammation.

There is an increasing understanding of the roles of proresolving lipid mediators in treating infection. As a new family of anti-inflammatory and proresolving lipid mediators, it has been previously confirmed that maresins limit the further recruitment of PMNs and inhibit neutrophil infiltration in vivo yet stimulate the nonphlogistic recruitment of mononuclear cells. When macrophages encounter maresins, they increase phagocytosis and efferocytosis, resulting in the removal of microbes. Biosynthesized maresins counterregulate the proinflammatory cytokines such as IL-1*β*, IL-6, and TNF-*α*. They also regulate nuclear factor kappa B (NF-*κ*B) gene products and increase the regulation of T cell de novo synthesis and intracellular levels of cyclic adenosine monophosphate, regenerate tissue, and play a role in antinociceptive action [9, 29] (Figure 2).

## 5. Maresins in Lung Disease

Acute inflammation is a form of innate immune defense and is the primary response to injury and infection. In the lungs, dysregulated acute inflammation and failure to resolve inflammation are the major contributors of numerous lung diseases, which can result in lung injury, contributing to pulmonary fibrosis that severely impairs essential gas exchange processes [26].

IL-6 is a pleiotropic cytokine best recognized as a primary mediator of the acute phase response [30]. IL-6 not only activates neutrophils but also delays the phagocytosis of macrophages in acute inflammation, which can promote a “cytokine storm.” A number of stimuli, including inflammatory cytokines and growth factors, such as TNF-*α*, IL-1, and platelet-derived growth factor (PDGF), are associated with increases in vascular cell-derived IL-6 [31, 32]. IL-6, IL-1, and TNF-*α* are all sensitive indicators of inflammatory reaction, which can reflect the condition of patients and evaluate the severity of inflammatory reaction. By early monitoring of these important indicators, we can take appropriate measures to stop the further development of the inflammatory response. IL-6 can play a positive role in some specific aspects of lung disease. Inhibition of IL-6 (or IL-6R) may be a therapy for asthma, chronic obstructive pulmonary diseases (COPD), and other lung diseases.

Maresin 1 as a specialized proresolving lipid mediator has been shown to reduce airway inflammation associated with acute and repetitive exposure to organic dust by activating protein kinase C (PKC) isoforms *α* and *ε* [33], limiting neutrophil infiltration, and decreasing IL-6, TNF-*α*, and chemokine C-X-C motif ligand 1 levels, which suggests that maresin 1 could contribute to an effective strategy for reducing airway inflammatory diseases induced by agricultural-related organic dust environmental exposure [34]. 100 nmol/L maresin 1 can attenuate the proinflammatory cytokines (TNF-*α*, IL-1*β*, and IL-6), chemokines, pulmonary myeloperoxidase activity, and neutrophil infiltration in an LPS-induced acute lung injury (ALI) mouse and can significantly inhibit LPS-induced ALI by restoring oxygenation, attenuating pulmonary edema, and mitigating pathohistological changes [35]. This study also shows that maresin 1 exhibits novel mechanisms in the resolution of inflammation in that it can inhibit proinflammatory mediator production by LTA4 hydrolase and can block arachidonate conversion by human 12-LOX, rather than merely terminating phagocyte involvement [20]. Furthermore, maresin 1 can also maintain the permeability of lung epithelial cells by upregulating the expression of claudin-1 and Zonula occludens protein 1 (ZO-1) in ALI [36].

Recently, metabololipidomics of murine lungs identified temporal changes in endogenous maresin 1 during self-limited allergic inflammation. Exogenous maresin 1 augmented de novo generation of regulatory T cells (Tregs), which interacted with innate lymphoid cells (ILC2s) to markedly suppress cytokine production in a transforming growth factor *β*1- (TGF-*β*1)-dependent manner, suggesting the use of maresin 1 as the basis for a new proresolving therapeutic approach in asthma and other chronic inflammatory diseases [37]. In addition, the study also found that treating mouse type II alveolar epithelial cells with maresin 1 significantly prevented TGF-*β*1-induced fibronectin and alpha-smooth muscle actin (*α*-SMA) expression and restored E-cadherin levels in vitro, as well as attenuating bleomycin-induced lung fibrosis in vivo [38]. These studies suggest that maresin 1 can be used as a promising new strategy for treating lung inflammation-related diseases.

## 6. Maresins in Vascular Disease

Vascular injury induces a potent inflammatory response that influences vessel remodeling and patency, limiting the long-term benefits of cardiovascular interventions such as angioplasty. Inflammatory resolution is central to vascular repair. Chatterjee et al. [14] confirmed that maresin 1 imparted a strong anti-inflammatory phenotype in human vascular smooth muscle cells and endothelial cells, associated with reduced monocyte adhesion and TNF-*α*-induced production of reactive oxygen species (ROS) and NF-*κ*B activation by inhibiting I*κ*B kinase (IKK) phosphorylation, NF-kappa-B inhibitor alpha (I*κ*B-*α*) degradation, and nuclear translocation of the NF-*κ*B p65 subunit. Maresin 1 also inhibited mouse aortic smooth muscle cell migration, relative to a PDGF gradient, and reduced TNF-*α*-stimulated p65 translocation, superoxide production, and proinflammatory gene expression. In vivo, maresin 1 reduced neutrophil and macrophage recruitment and increased polarization of M2 macrophages in the arterial wall [39]. These results offer new opportunities to regulate the vascular injury response and promote vascular homeostasis. In addition, research has shown, for the first time, that human platelets express the SPM receptors G-protein-coupled receptor 32 (GPR32) and ALX, and maresin 1 regulates platelet hemostatic function by enhancing platelet aggregation and spreading, while suppressing the release of proinflammatory and prothrombotic mediators, indicating maresin 1 could be a novel class of antiplatelet agents that play an important role in the resolution of inflammation in cardiovascular diseases [40].

## 7. Maresins in Metabolic Disease

Chronic low-grade inflammation associated with metabolic diseases is sustained and detrimental. SPMs can stop and limit further PMN entry and stimulate macrophage intake and clearance of apoptotic cells, debris, and bacteria; treatment with specific SPMs improves metabolism and immunity [28]. Viola et al. [41] found that maresin 1 prevented atheroprogression by inducing a shift in macrophage profile toward a reparative phenotype and stimulated collagen synthesis in smooth muscle cells. Recently, a study has confirmed that maresin 1 reduced the expression of MCP-1 (monocyte chemotactic protein 1), TNF-*α*, IL-1*β*, and the proinflammatory M1 macrophage phenotype marker Cd11c, while it upregulated adiponectin and glucose transporter-4 protein (Glut-4) and increased protein kinase B (Akt) phosphorylation in white adipose tissue (WAT) in diet-induced obese (DIO) mice; maresin 1 also improved the insulin tolerance test and increased adiponectin gene expression, Akt and adenosine monophosphate-activated protein kinase (AMPK) phosphorylation, and the expression of M2 macrophage markers Cd163 and IL-10 in genetic (ob/ob) obese mice [42]. Our previous research showed that maresin 1 may have a protective effect on diabetic nephropathy by mitigating the expression of the NLRP3 inflammasome, TGF-*β*1, and fibronectin (FN) in mouse glomerular mesangial cells [43]. Furthermore, Hong et al. [44] found that maresin-like mediators (14,22-dihydroxy-docosa-4Z,7Z,10Z,12E,16Z,19Z-hexaenoic acids) were produced by leukocytes and blood platelet (PLT) and were involved in wound healing by restoring reparative functions to diabetic macrophages; in addition, these mediators could ameliorate the inflammatory activation of macrophages and had the potential to suppress chronic inflammation in diabetic wounds caused by the activation of macrophages. Resolution of inflammation may be an essential criterion in developing future therapeutic interventions aimed at counteracting inappropriate inflammation in metabolic disease.

## 8. Maresins in Inflammatory Bowel Disease

The gut is regarded as being in a state of controlled inflammation; resolution of inflammation is thus critical to avoid excessive damage to host tissue. It has been previously reported that maresin 1 consistently protects mice in models of experimental colitis by inhibiting the NF-*κ*B pathway and consequently multiple inflammatory mediators, such as IL-1*β*, TNF-*α*, IL-6, and porcine interferon *γ* (IFN-*γ*), while enhancing the macrophage M2 phenotype [45]. Recently, Wang et al. [46] found that maresin 1 treatment ameliorated iron-deficient anemia by reducing colonic inflammation and inhibiting hepcidin expression though the IL-6/STAT3 pathway. In addition, maresin 2 showed the potential anti-inflammatory action in mouse peritonitis initiated by intraperitoneal injection of zymosan. This study found that maresin 2 is equivalent to maresin 1 in limiting neutrophil infiltration, whereas maresin 1 is more effective in enhancing macrophage phagocytosis than maresin 2 [15]. Current studies on maresin 2 are still limited and require additional experiments to explore its biological effects and mechanisms.

## 9. Maresins Stimulate Tissue Regeneration and Control Pain

Acute inflammatory responses are protective, and the cardinal signs of inflammation are heat, redness, swelling, and eventual loss of function. Proresolving mediators have been shown to be the stop signals of inflammation and act in the host defense mechanism to reduce pain and enhance wound healing and tissue regeneration [39]. Transient receptor potential V1 (TRPV1) was found to be expressed in primary sensory neurons and plays an important role in mediating heat pain and heat hyperalgesia after injury [47]. Serhan et al. [47] have confirmed that maresin 1 dose-dependently inhibited TRPV1 currents in neurons, blocked capsaicin-induced inward currents, and reduced both inflammation-induced and chemotherapy-induced neuropathic pain in mice. Meanwhile, maresin 1 markedly reduced vincristine-induced mechanical allodynia and accelerated surgical regeneration in planaria, increasing the rate of head reappearance. Recently it was reported that macrophages produce a family of bioactive peptide-conjugated mediators known as maresin conjugates in tissue regeneration (MCTR) [16]. These mediators have been found to rescue *Escherichia coli* infection-mediated delay in tissue regeneration in planaria and were shown to protect mice from second-organ reflow injury, promoting repair by limiting neutrophil infiltration, upregulating nuclear antigen KI-67, and roof plate-specific spondin 3 [16]. To assess the ability of each synthetic MCTR to promote tissue regeneration in planaria, one study found that each of the three synthetic MCTRs dose-dependently (1–100 nM) accelerated tissue regeneration in planaria by 0.6–0.9 days; MCTR3 and MCTR2 were more potent than MCTR1. In mice, MCTRs were also found to regulate tissue repair and regeneration in lung tissue where administration of their key enzymes during ischemia-reperfusion-mediated injury protected the lung from leukocyte-mediated damage and upregulated the expression of molecules that are associated with cell proliferation and tissue repair in the lung [16]. Furthermore, each MCTR promoted resolution of *E*. *coli* infections in mice by increasing bacterial phagocytosis, limiting neutrophil infiltration, and promoting efferocytosis [48]. Therefore, these results demonstrate the potent actions of maresins in regulating inflammation resolution, tissue regeneration, and pain resolution.

## 10. Conclusion and Prospects

Maresins are part of the latest families of anti-inflammatory lipid mediators, which display both anti-inflammatory and proresolving activities in acute or chronic inflammatory-related diseases. Maresins are synthesized by the lipoxygenase enzyme oxidation pathway during the inflammation-subsiding period and conjugate triene double bonds. Studies have confirmed that maresins protect the body by limiting neutrophil infiltration, enhancing macrophage phagocytosis, reducing the production of proinflammatory factors, inhibiting NF-*κ*B activation, stimulating tissue regeneration, and controlling pain. Therefore, maresins as potent inflammatory self-limiting factors are expected to become highly promising anti-inflammatory intervention drug targets. And as inflammation is closely related to fibrosis, studying maresin may also provide new directions for the prevention and treatment of viscera fibrosis. In addition, further investigations are required to understand the relationship between novel endogenous pathways to control pathogens and microbial pathogenesis diversity. We envisage more basic research and clinical research on maresins. We also expect to discover maresin-related stable analogues or new family members of specialized proresolving lipid mediators as potential reserve molecules for exploiting endogenous anti-inflammatory mechanisms to limit excessive pathogen-mediated inflammatory responses in future therapeutic strategies.

## Figures and Tables

**Table 1:** Classification and structure of maresins.

Designation	Chemical structures	Key enzyme	Bioactions and function
Maresin 1	7R,14S-Dihydroxy-docosa-4Z,8E,10E,12Z,16Z,19Z-hexaenoic acid [18]	12-Lipoxygenase, epoxide hydrolysis [49]	Limits PMN infiltration [50]; enhances macrophage phagocytosis and efferocytosis [51]; shortens resolution interval and suppresses oxidative stress [52]; counterregulates proinflammatory chemical mediators [53]; controls pain and enhances tissue regeneration [47]
Maresin 2	13R,14S-Dihydroxy-4Z,7Z,9E,11E,16Z,19Z-hexaenoic acid [54]	12-Lipoxygenase, soluble epoxide hydrolase [54]	Limits PMN infiltration; enhances macrophage phagocytosis [54, 55]
MCTR1	13R-Glutathionyl,14S-hydroxy-4Z,7Z,9E,11E,13R,14S,16Z,19Z-docosahexaenoic acid [16]	12-Lipoxygenase, leukotriene C4 synthase, and glutathione S-transferase MU 4 [53, 56]	Stimulates tissue regeneration and reduces neutrophil infiltration: MCTR3 ≈ MCTR2 > MCTR1 Shortens resolution interval (Ri) : MCTR2 > MCTR3 > MCTR1 Regulates local eicosanoids during infections: MCTR1 > MCTR3 > MCTR2 Enhances macrophage phagocytosis: MCTR3 > MCTR1 > MCTR2 [9, 17, 56]
MCTR2	13R-Cysteinylglycinyl,14S-hydroxy-4Z,7Z,9E,11E,13R,14S,16Z,19Z-docosahexaenoic acid [56]	12-Lipoxygenase, gamma-glutamyltransferase [53, 56]	
MCTR3	13R-Cysteinyl,14S-hydroxy-4Z,7Z,9E,11E,13R,14S,16Z,19Z-docosahexaenoic acid [17]	12-Lipoxygenase, dipeptidase [53, 56]	

## Abbreviations

SPMs: Specialized proresolving lipid mediators
DHA: Docosahexaenoic acid
PMNs: Polymorphonuclear leukocytes
Ri: Resolution interval
EPA: Eicosapntemacnioc acid
MCTR: Maresin conjugate in tissue regeneration
sEH: Soluble epoxide hydrolase
12-LOX: 12-Lipoxygenase
LTC4S: Leukotriene C4 synthase
GSTM4: Glutathione S-transferase MU 4
GGT: Gamma-glutamyltransferase
DPEP: Dipeptidase
NF-*κ*B: Nuclear factor kappa B
PDGF: Platelet-derived growth factor
TGF-*β*1: Transforming growth factor *β*1
TRPV1: Transient receptor potential V1.
